# Dynamics of levitated objects in acoustic vortex fields

**DOI:** 10.1038/s41598-017-07477-1

**Published:** 2017-08-02

**Authors:** Z. Y. Hong, J. F. Yin, W. Zhai, N. Yan, W. L. Wang, J. Zhang, Bruce W. Drinkwater

**Affiliations:** 1 Department of Applied Physics, Northwestern Polytechnical University, Xi’an, 710072 China; 2 Department of Mechanical Engineering, University Walk, University of Bristol, Bristol, BS8 1TR United Kingdom

## Abstract

Acoustic levitation in gaseous media provides a tool to process solid and liquid materials without the presence of surfaces such as container walls and hence has been used widely in chemical analysis, high-temperature processing, drop dynamics and bioreactors. To date high-density objects can only be acoustically levitated in simple standing-wave fields. Here we demonstrate the ability of a small number of peripherally placed sources to generate acoustic vortex fields and stably levitate a wide range of liquid and solid objects. The forces exerted by these acoustic vortex fields on a levitated water droplet are observed to cause a controllable deformation of the droplet and/or oscillation along the vortex axis. Orbital angular momentum transfer is also shown to rotate a levitated object rapidly and the rate of rotation can be controlled by the source amplitude. We expect this research can increase the diversity of acoustic levitation and expand the application of acoustic vortices.

## Introduction

The nonlinearity of acoustic wave propagation in liquid and gaseous media is known to generate acoustic radiation forces^1^ which have been harnessed to create fluid flow^2^, microparticle manipulation in liquids^3^ and to manipulate larger objects suspend in gases^4–6^. Using acoustic radiation forces to achieve levitation is of significant practical importance as it removes the need for the levitated object to be in contact with surfaces^7^ and hence been used widely in containerless processing^8–10^, chemical analysis^11, 12^, drop dynamics^13, 14^ and bioreactors^15, 16^.

In the most common configuration for airborne acoustic levitation is a single axis levitator is used in which a standing-wave field is formed between a sound emitter and a reflector, separated by an appropriate distance^17^. Objects which are denser and/or less compressible than the host fluid can be trapped in the nodes of acoustic pressure, which are spaced at half-wavelength intervals. The pressure nodes correspond to minima in potential energy and are hence often referred to as potential-wells. For example, high-density media such as liquid mercury and solid iridium can be levitated^18^ and high-temperature alloys can be containerlessly processed in these devices^10^. However, the acoustic field in single axis levitators produces relatively large axial acoustic radiation forces and relatively weaker lateral forces, leading to poor lateral trapping stability. If liquid droplets are levitated in such systems they are often distorted into flat discs and unstable surface ripples are generated, both of which lead to atomization^19^. In addition the presence of the transducers and reflections in a single axis configuration limits the range of observation angles.

Various levitator configurations have recently been suggested to overcome some of the above limitations of single-axis systems. Foresti *et al*. employed multiple piezoelectric resonators to move drops and particles laterally in air by spatiotemporal modulation of the acoustic field^20, 21^. Zhang *et al*. showed that by controlling the phases of the elements in a 40-transducer planar array, bottle beams could be used to create a three dimensional potential-well with a uniform force distribution^22^. Marzo *et al*. showed how the drive signals to 50–100-trasnducer arrays could be optimized to create three dimensional manipulation and demonstrated the controlled translation and slow rotation of low density objects^23^. In addition Andrade *et al*.^24^ used three transducers placed in close proximity around a large light sphere and achieved levitation by setting up quasi-one-dimensional standing waves between each transducer and the surface of the sphere.

Acoustic vortices^25–27^ are acoustic waves which have helicoidal wavefronts and null-pressure centres and carry orbital angular momentum similar to that of optical vortices^28, 29^. Vortex-field acoustic levitation has recently been demonstrated experimentally both in water and air using large arrays of transducers and shown to have the ability to control particle trapping and rotation simultaneously^23, 27, 30^ which should be due to the coexistence of acoustic radiation force and torque^31–35^. However, in air, only a few experiments have been published and these have all concerned very low density solid objects (i.e. expanded polystyrene, *ρ* = 20–50 kg/m^3^). A dynamic instability and ejection from the trap has been observed^23^.

In this paper, a range of new three-dimensional first order Bessel function shaped potential-well structures of arising from acoustic vortex fields are explored. Here, a small number of peripherally placed sources are used to dramatically simplify the experimental configuration. This apparatus is then shown to generate airborne acoustic vortices and stably levitate a range of liquids and solids. The acoustic radiation forces exerted on spherical objects in these acoustic vortex fields are predicted and the stable oscillatory and rotational dynamic behavior of levitated water droplets and various solid matters are observed experimentally.

## Results

### Potential-well structures

Acoustic vortices are characterized by an azimuthal phase, *θ*, dependence of the form *e* ^−*imθ*^, where the integer *m* is the order or topological charge of the helicoidal beam and this encodes the angular rotation rate of the wavefront^25^. Acoustic vortices can be achieved by employing an array with the source elements excited by sinusoidal voltages, delayed by appropriate phases^32, 33^. Here we use arrays, shaped as regular polygons, with three to six peripherally distributed sources (Supplementary Fig. S1) and a 2π*m* phase ramp applied to the drive signals (see Methods).

**Figure 1:**
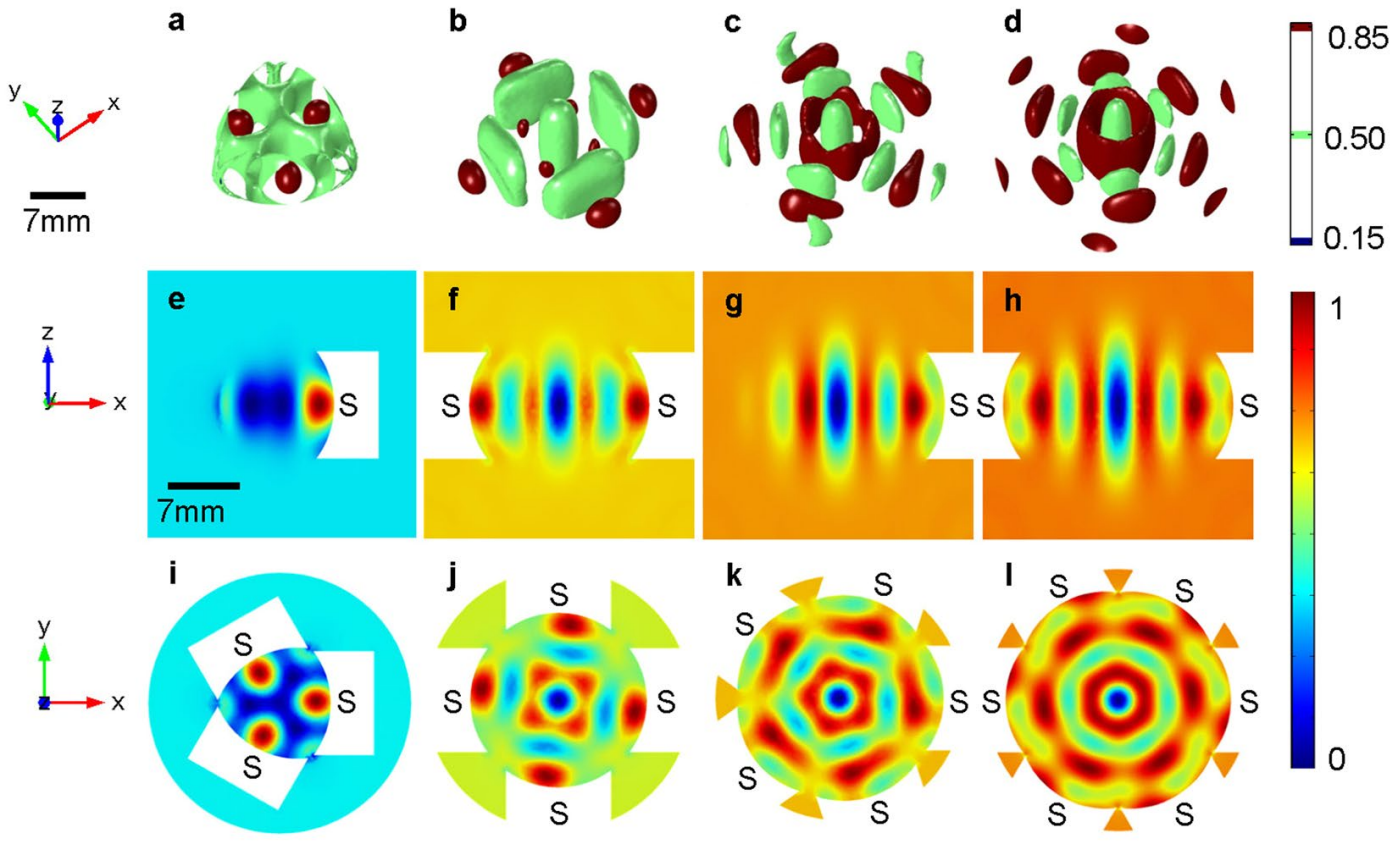
Potential-well structures of first-order acoustic vortex fields generated by three to six peripherally placed acoustic sources. The symbol “S” denotes an acoustic source. (**a**–**d**) The potential-well structures are displayed as three-dimensional isosurfaces of the normalized Gor’kov potential, (**e**–**h**) normalized Gor’kov potential in *x*-*z* plane and (**i**–**l**) *x*-*y* plane.

To characterize the trap positions and stability of the acoustic vortex fields, the Gor’kov potential^36^ is calculated (see Methods) and the results shown in Fig. 1. It can be seen that the regular polygon arrangements all create three-dimensional potential wells which are elongated along the device axis (i.e. *z* = 0). With an increased number of sources (and setting the helicoidal order, *m* = 1) the acoustic potential field in the *x*-*y* plane tends to that of a first-order Bessel-shape which is axisymetric^37^ (see Supplementary Fig. S2).

**Figure 2:**
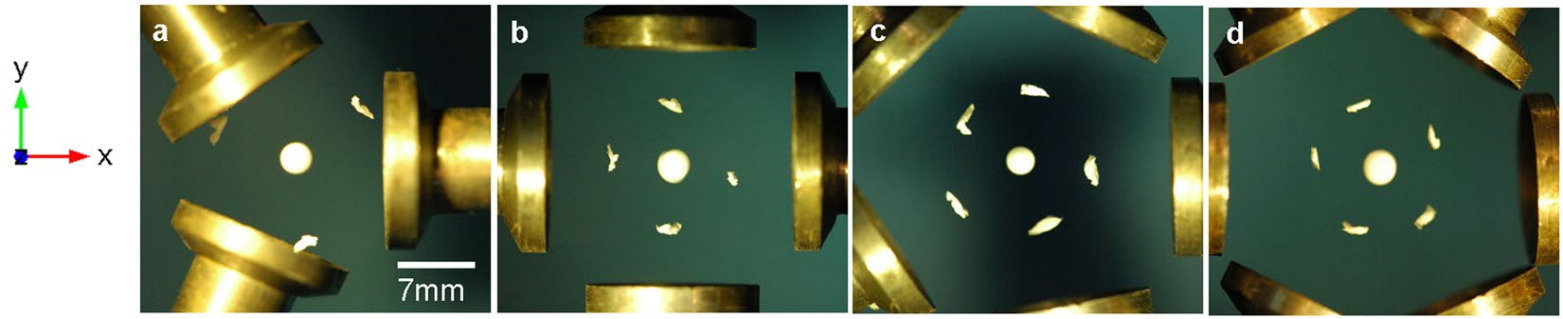
Photographs (top view) of levitated expanded polystyrene objects used to visualize the experimentally achieved potential-well structures.

To examine the potential-well structures, the levitation experiments were performed (see Methods) and the results are shown in Fig. 2. From Figs 1 and 2 it can be seen that, due to the finite number of transducers used here, around each axially located potential-well, there are number of shallow satellite potential minima. Our simulations suggest that the number of satellite minima should equal the number of sources, however, as shown in Fig. 2 this was not observed beyond 5 transducers, which we attribute to the formation of small parasitic potential-wells due to transducer inconsistencies. However, for 3–5 transducers the positions of the trapped expanded polystyrene objects show a good agreement with the predicted positions of the minima potential (Fig. 1). The following sections now consider the stability of the axially located potential-well with a focus on the 3 and 4 transducer systems.

### Acoustic radiation force

**Figure 3:**
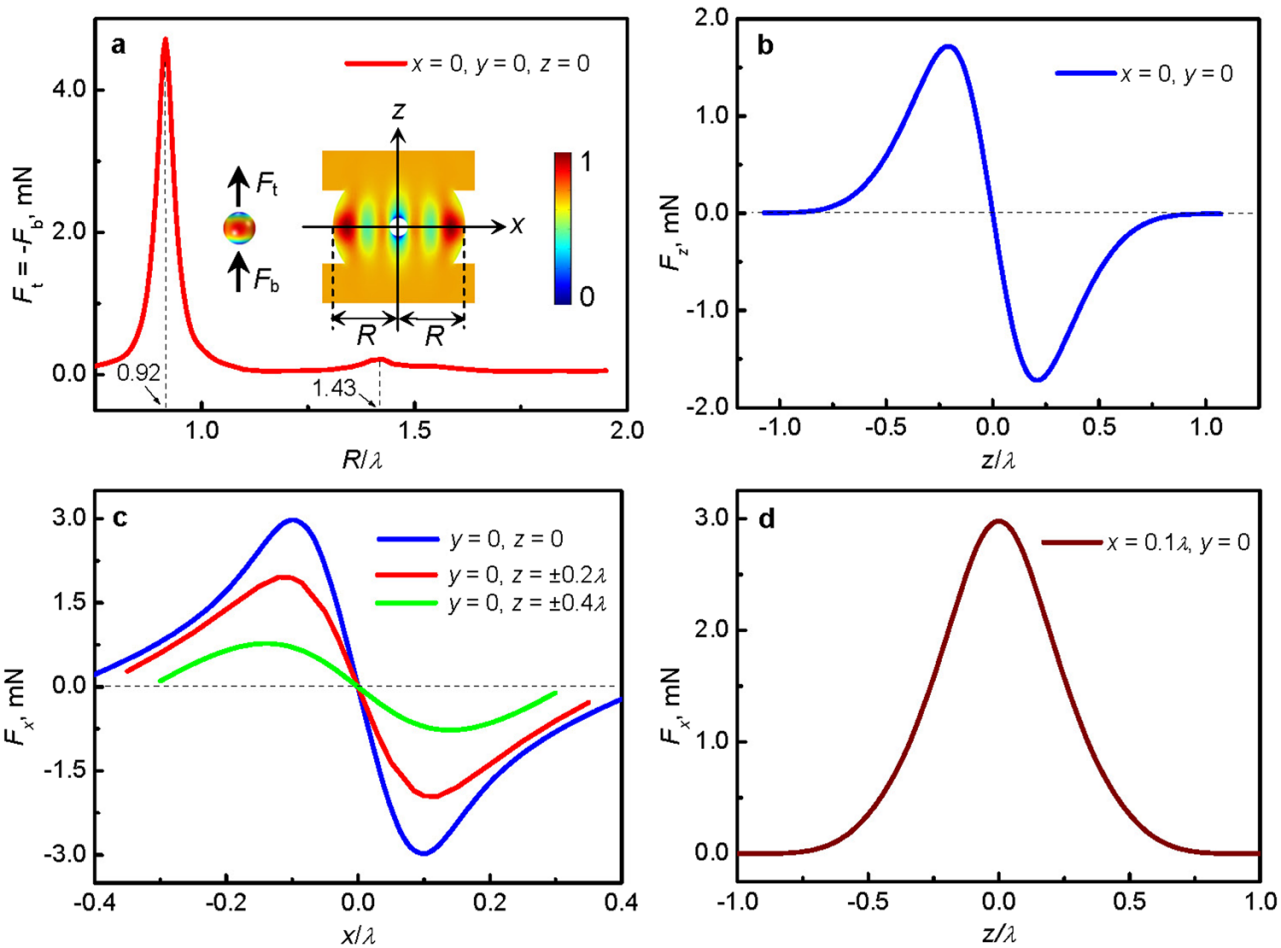
Simulation of the acoustic radiation force on a rigid sphere in acoustic vortex field. Results are for the 4-transducer arrangement and a rigid sphere of radius 1.5 mm. (**a**) Effect of device dimension, *R*, on the vertical component of acoustic radiation force obtained by respectively integrating acoustic radiation pressure over the top (*z* > 0) hemisphere (*F*_t_) and bottom (*z* < 0) hemisphere (*F*_b_) of a rigid sphere located at (0,0,0). Using *R* = 0.92*λ*, the insert shows the distribution of the normalized Gor’kov potential in the *x*-*z* plane. (**b**) *z*-component of the total force on the sphere as a function of location on the *z*-axis. (**c**) The *x*-component of force on the sphere as a function of location on the *x*-direction. (**d**) The *x*-component force on the sphere as a function of location on the line (0.1*λ*,0,*z*).

The acoustic radiation force on a rigid sphere (radius, 1.5 mm) in an acoustic vortex field generated by 4 transducers is now discussed (see Methods for details of the model used). Figure 3a shows the acoustic radiation forces exerted on the top (*z* > 0) hemisphere (*F*_t_) and bottom (*z* < 0) hemisphere (*F*_b_) when the sphere is placed centrally, as a function of transducer separation. The forces can be seen to vary dramatically with transducer separation due to the resonant behavior of the central cavity, which has its first mode at, *R* = 0.92*λ* and its second at *R* = 1.43*λ*. We note also that, away from the resonant conditions, the forces decrease with increasing separation. Given these findings, we built transducers arrays in the first resonant condition (*R* = 0.92*λ*), where the input electrical energy is most efficiently converted to radiation forces. Note that the forces exerted on the top and bottom hemispheres are in the z-direction and directed away from the vortex centre (*F*_t_ > 0 and *F*_b_ < 0). This means that a deformable object in the central trap will be stretched along the vortex axis, namely the *z*-axis. At first glance this apparently tensile acoustic radiation pressure appears non-physical as it arises from a pressure which must always be compressive. However, the contradiction is removed because the radiation pressure is an *excess pressure* (see Methods) meaning that the forces are always compressive, the negative value meaning that the radiation pressure is less than that of the atmosphere. More discussion and application of tensile acoustic radiation pressure can be found elsewhere in refs 38 and 39.

The *z*-component of the total force (*F*_*z*_ = *F*_t_ + *F*_b_) exerted on a rigid sphere is then calculated as a function of position along the vortex axis and the result is shown in Fig. 3b. Here it can be seen that *F*_*z*_ is always towards the potential-well centre and so the trapping is stable in the *z*-direction. However, it should be noted that in reality, an object will be levitated at a point below the central point where the radiation force and gravity are balanced. The lateral (horizontal) force (*F*_*x*_) is investigated by positioning the sphere away from the vortex axis and the results are shown in Fig. 3c and d. It is found that, in the central region, the lateral force points towards the vortex axis and the magnitude of *F*_*x*_ (or *F*_*y*_) is larger than *F*_*z*_ meaning that the trapping in the central trap is stable in all three dimensions.

### Vortex-field acoustic levitation

**Figure 4:**
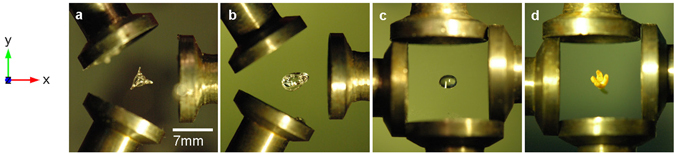
Levitating dense objects in acoustic vortex fields (top view). (**a**–**d**) The levitated objects are silicone oil, silicone bubble, water droplet and *Chimonanthus* (Wintersweet) flower, respectively. Note that the array with three transducers is used for (**a** and **b**) and the array with four transducers for (**c** and **d)**.

Based on the simulations, the separation of the transducers in the arrays with three and four piezoelectric transducers is set to *R* ≈ 0.9*λ* (and frequency, *f* = 36.4 kHz) and used to levitate objects such as silicone oil and water droplets as well as a *Chimonanthus* flower, as shown in Fig. 4 (see Supplementary Movies 1–3 and note that the levitation of additional objects is shown in Supplementary Fig. S3). In Fig. 4a, the levitated silicone oil droplet is shaped as convex pseudo-triangle coinciding well with the potential-well structure shown in Fig. 1a. In Fig. 4c and Supplementary Fig. S4, the equilibrium shapes of the water droplets are shown to be ellipsoidal with the major axis aligned with the vortex axis. Figure 4d shows a levitated *Chimonanthus* flower which stabilizes in a position such that its stem is aligned with the vortex axis (Supplementary Fig. S3c and d, show a levitated seedling with an elongated root and a twig which also align with the vortex axis).

### Dynamic behavior of the levitated water droplets

**Figure 5:**
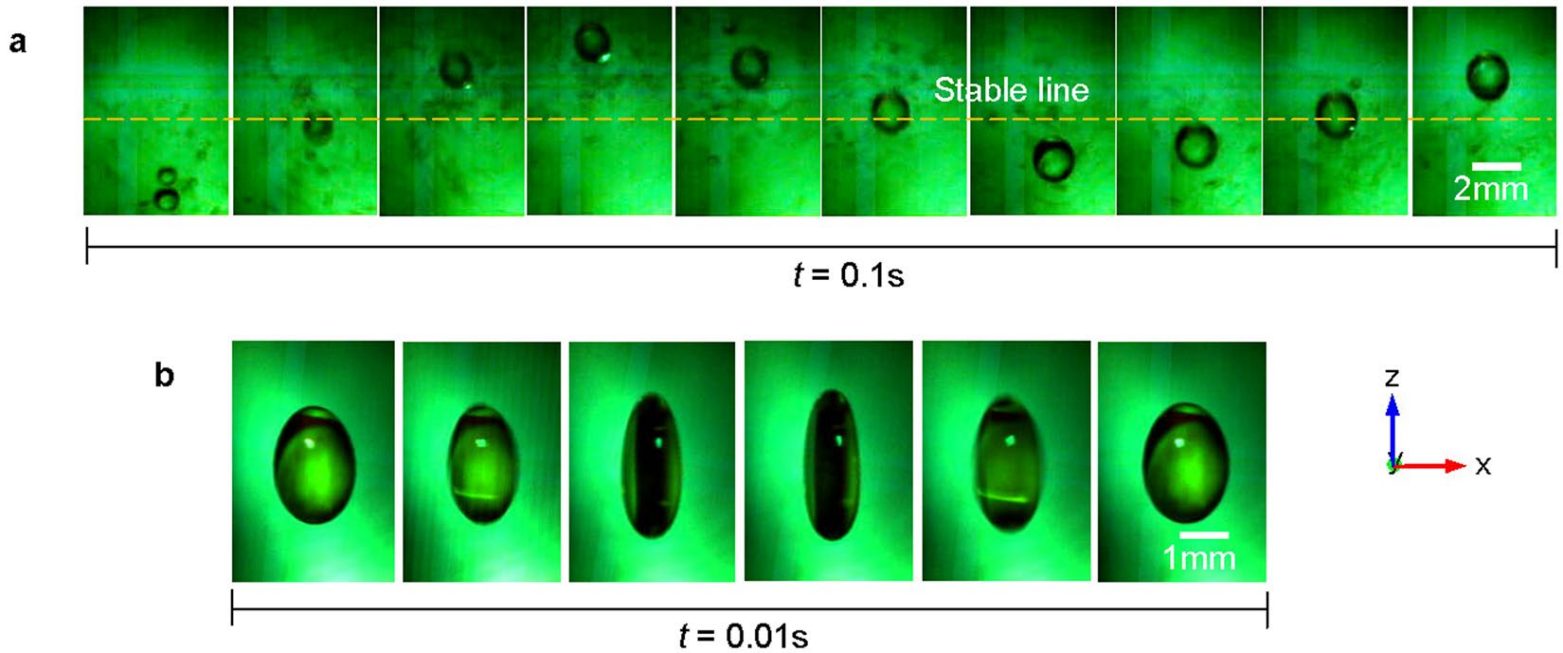
Drop oscillation in acoustic vortex field in air. (**a**) The drop formation process and the vertical drop oscillation in the central trap. The dashed line across the images gives the stable height of the final drop. (**b**) Drop shape oscillation in the central trap. The vortex field is generated by four transducers.

The dynamic behavior of the levitated water droplets was observed in the acoustic vortex field generated by four peripherally placed transducers arranged at *R* ≈ 0.9*λ* (see Methods). Firstly, sub-millimeter water droplets are injected just below the central trap and are caused to accelerate upwards towards the potential-well centre, as shown in Fig. 5a and Supplementary Movie 4. Initially multiple water droplets are present which quickly coalesce into a single millimeter-sized droplet which oscillates with decreasing amplitude until it stabilizes at an equilibrium position where the radiation force and gravity balance. The vertical vibration frequency *f* of the final droplet is ≈15 Hz. From Fig. 3b, the gradient of the force-distance curve near the array centre is near-linear. Therefore, the trapping performance can be quantified by a stiffness which can be extracted by assuming the object-trap system to be a simple mass-spring oscillator. Here, the radius of the final water droplet is about 0.7 mm, leading to a droplet mass *m* of 1.4 × 10^−6^ kg, and from, $2{\rm{\pi }}f=\sqrt{\frac{k}{m}}$, the stiffness *k* can be calculated as 0.013 N/m. This compares well with the value of 0.012 N/m obtained from the simulation, i.e. the gradient of Fig. 3b at *z* = 0.

The shape oscillation about the equilibrium shape of the centrally trapped water droplet is shown in Fig. 5b (and Supplementary Movie 5), from which it can be seen that the major axis of the droplet oscillates by a stretching and receding along the vortex axis. The frequency of the shape oscillation is ≈100 Hz. We note that similar resonant effects have been extensively studied in single-axis levitators^12, 13, 40–42^. The rotation of the centrally trapped water droplet due to acoustic orbital angular momentum (OAM) transfer can also be observed in Supplementary Movie 5. However, because of the combined motion of oscillation and rotation, the rotation rate of the water droplet is difficult to determine.

**Figure 6:**
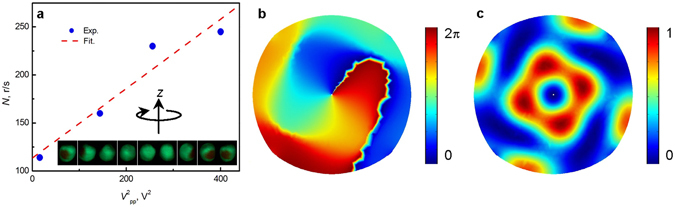
High-speed spinning of an expanded polystyrene particle in acoustic vortex field. The vortex field is generated by four transducers. The particle of radius 1.5 mm is levitated in the central trap of the acoustic vortex field. (**a**) The experimental rotation rate of the particle as a function of the voltage applied to drive the transducers. Rotation measured using a high speed camera (see Methods). (**b**) The simulated phase distribution of the acoustic field in the *x*-*y* plane. (**c**) The simulated normalized density distribution^26^ of the orbital angular momentum in the *x*-*y* plane.

Figure 6a shows how the rotation rate of an expanded polystyrene particle varies with applied signal voltage (also see video in Supplementary Movie 6). It was found that the rotation rate increases with the square of the voltage applied to the transducers and the largest rotation rate exceeds 250 revolutions per second (i.e. r/s). This relationship suggests that the torque is proportional to the acoustic power^32–35^, which is in agreement with observations in water^26, 27^. We note that the OAM transfer is thought to be governed by the absorption of sound by the fluid and the rotation speed by the viscosity of the fluid^27^. Comparing the water-borne case to the airborne, the absorption is lower by 3 orders of magnitude and the viscosity is higher by 2 orders of magnitude. Our observations therefore suggest that, despite the large differences in these physical properties, the same phenomena exist. The high energy concentrated in the central region overcomes the low efficiency of the OAM transfer from air to the levitated particle in our system. At this highest rotation rate, a whistling sound is emitted by the spinning particle which can be heard by a listener located near the apparatus (see videos in Supplementary Movies 7 and 8). We note that the dominant frequency of the emitted sound coincides with the rotation rate which leads to the possibility of using a simple acoustic measurement to determine rotation rate. The phase distribution and the density distribution of the orbital angular momentum at the central area are plotted in Fig. 6b and c. It is shown that the centre of the central trap is a phase singularity and the orbital angular momentum distributes around this axial phase singularity. Hence, it is shown that the orbital angular momentum transfer also takes place in air, just as has been observed in water^26, 27, 33^. Note that the rotation direction and rate can be controlled by adjusting the direction and the energy density of orbital angular momentum.

## Discussion

Acoustic levitation of objects in air is more challenging than that in water because of the lower transduction efficiency and the significantly lower buoyancy force in air. Single-axis acoustic levitators typically use a sound emitter and reflector, which are usually aligned parallel to the gravity direction and hence block the observation of the levitated objects from top and bottom^18^. In such levitators, liquid droplets are often flattened into disc-like shapes^19^ and rapidly atomizatized^43^. The dynamic behavior of particles in a Bessel shaped field are not simple as unlike the microfluidic cases considers to date^27^ mass and inertia play an important role. Further complexity comes from the fact that the restoring forces vary spatially and the particles spin at high speeds due to the low viscosity of the air. However, despite this complexity we show that our airborne acoustic vortex fields result in relatively simple oscillatory dynamics and this enables controlled and stable levitation and rotation of a range of liquids and various solid objects in air. There are four main conclusions from this paper. (i) Acoustic vortices capable of stably trapping objects in three-dimensions can be created by a relatively low number of transducers. This simplification will bring this levitation capability within the reach of a wide range of new researchers. (ii) An elongated object in an airborne acoustic vortex field tends to stand upright with its long axis coincident with the vortex axis. This has potential applications in container-less processing. (iii) A liquid droplet in an airborne acoustic vortex field can be stretched along the vortex axis due to the non-uniform distribution of forces. (iv) Using these simplified array devices, an object levitated in air can be controllably spun, due to orbital angular momentum transfer from the acoustic wave to the levitated object. This capability also has the potential to open up new applications in non-contact manipulation of matter in air.

## Methods

### Generation of acoustic vortex fields

In simulation, three-dimensional acoustic models of the regular-polygon arrays with three to six peripherally and uniformly distributed acoustic sources were built by employing finite element analysis (Comsol Multiphysics, Acoustics module). Each source has a radius of 7 mm and is spherically concaved with a curvature radius of 10 mm. Each source is individually driven by sinusoidal signals which are phase-shifted such that there is a ramp of 2*m*π phase delay around the array, i.e., the relative phase, *ϕ*_*n*_ of the signal applied to each of the N sources, *n* = 1, 2, 3, …, N, is given by^27^

1 $${\varphi }_{n}=\frac{2m\pi (n-1)}{{\rm{N}}}.$$

In the simulation, the frequencies of all the sinusoidal signals are set as 36.4 kHz and the amplitudes of all the sound emitting surfaces are given as 10 μm. Through this calculation, the acoustic pressure, *p*, and medium particle velocity, $\vec{v}$, can be obtained.

In experiment (see Supplementary Fig. S1), Langevin transducers which are actuated with piezoelectric elements, served as acoustic sources. The geometrical sizes of the sound emitting surfaces and the regular-polygon arrays were designed according to the geometrical parameters used in simulation. The operating conditions of the transducers was the same as in the simulation, i.e. frequency 36.4 kHz and the amplitudes of all the sound emitting surfaces were adjusted to be equal, around 10 μm.

### Evaluation of Gor’kov potential (force on a small sphere)

According to the theory of Gor’kov^36^, in an acoustic field, the acoustic radiation force exerted on a small spherical particle can be evaluated by, $\vec{F}=-\nabla U$, where *U* is the Gor’kov potential. The minimum positions of *U* in an acoustic field correspond to acoustic traps. The Gor’kov potential can be calculated from the simulated acoustic pressure and particle velocity as,

2 $$U=2\pi {a}^{3}[({\bar{p}}^{2}/3\rho {c}^{2}){f}_{1}-(\rho {\bar{v}}^{2}/2){f}_{2}]$$

where *a* is the radius of the spherical particle, ${\bar{p}}^{2}$ and ${\bar{v}}^{2}$ are the mean-square fluctuations of the acoustic pressure and particle velocity at the point where the particle is located and *ρ* and *c* are the density and sound speed of host medium, respectively. The acoustophoretic contrast factors *f*_1_ and *f*_2_ are given by ${f}_{1}=1-\rho {c}^{2}/{\rho }_{s}{c}_{s}^{2}$ and ${f}_{2}=2({\rho }_{s}-\rho )/(2{\rho }_{s}+\rho )$, where *ρ*_*s*_ and *c*_*s*_ are the density and sound speed of the particle. Here (i.e. Fig. 1) we evaluated the normalized Gor’kov potential, *U*/2*πa* ^3^ for a small rigid sphere, hence, *ρ*_*s*_ = ∞ and *f*_1_ = *f*_2_ = 1.

### Calculation of acoustic radiation force on a larger rigid sphere

The Gor’kov potential *U* obtained above can be used to evaluate the acoustic radiation force on a small rigid sphere. However, the method is not accurate for larger spheres (or other shapes) as it neglects the scattering induced by large particles. To predict the forces on larger particles (i.e. Fig. 3) we calculated the acoustic fields by including the rigid sphere in the finite element (Comsol) models and then obtained the acoustic radiation force, *F*, by solving the surface integral of the radiation pressure, *p*_*r*_, over the relevant surface. The excess acoustic radiation pressure, *p*_*r*_ is given by^44^

3 $${p}_{r}={\bar{p}}^{2}/2\rho {c}^{2}-\rho {\bar{v}}^{2}/2+{\rm{C}}$$

The constant C is given a finite value to satisfy any constraint on the system or is set to zero if there is no constraint (here C=0).

### Observation of the levitated objects

The pictures of acoustic levitation of diverse objects (Figs 2 and 4 and Supplementary Fig. S3) were taken by a Nikon D70s camera. The motion of the water droplets (Fig. 5, Supplementary Fig. S4, Supplementary Movies 4 and 5) and the expanded polystyrene particles (Fig. 6 and Supplementary Movie 6) in the acoustic vortex fields were recorded by a Redlake HG 100 K high-speed CCD with a frame rate adjustable between 500 and 2000 fps. The videos of acoustic levitation of diverse objects (Supplementary Movies 1–3, 7 and 8) were recorded by a Sony H400 camera.

## Electronic supplementary material

Supplementary Movie 1

Supplementary Movie 2

Supplementary Movie 3

Supplementary Movie 4

Supplementary Movie 5

Supplementary Movie 6

Supplementary Movie 7

Supplementary Movie 8

Supplementary Figures
