# Supplementary material for: Dynamics of levitated objects in acoustic vortex fields
Source: Sci Rep. 2017 Aug 2;7:7093. doi: 10.1038/s41598-017-07477-1 (PMC5540917; doi:10.1038/s41598-017-07477-1)
Supplement: Supplementary file 9 — Supplementary Figures [file 41598_2017_7477_MOESM9_ESM.doc]

**Supplementary Figures**

**Dynamics of levitated objects in acoustic vortex fields**

Z. Y. Hong^1,*^, J. F. Yin^1^, W. Zhai^1^, N. Yan^1^, W. L. Wang^1^, J. Zhang^2^ & Bruce W. Drinkwater^2^

^1^Department of Applied Physics, Northwestern Polytechnical University, Xi’an 710072, China.

^2^Department of Mechanical Engineering, University Walk, University of Bristol, Bristol BS8 1TR, United Kingdom.

^*^Correspondence to hongzy@nwpu.edu.cn


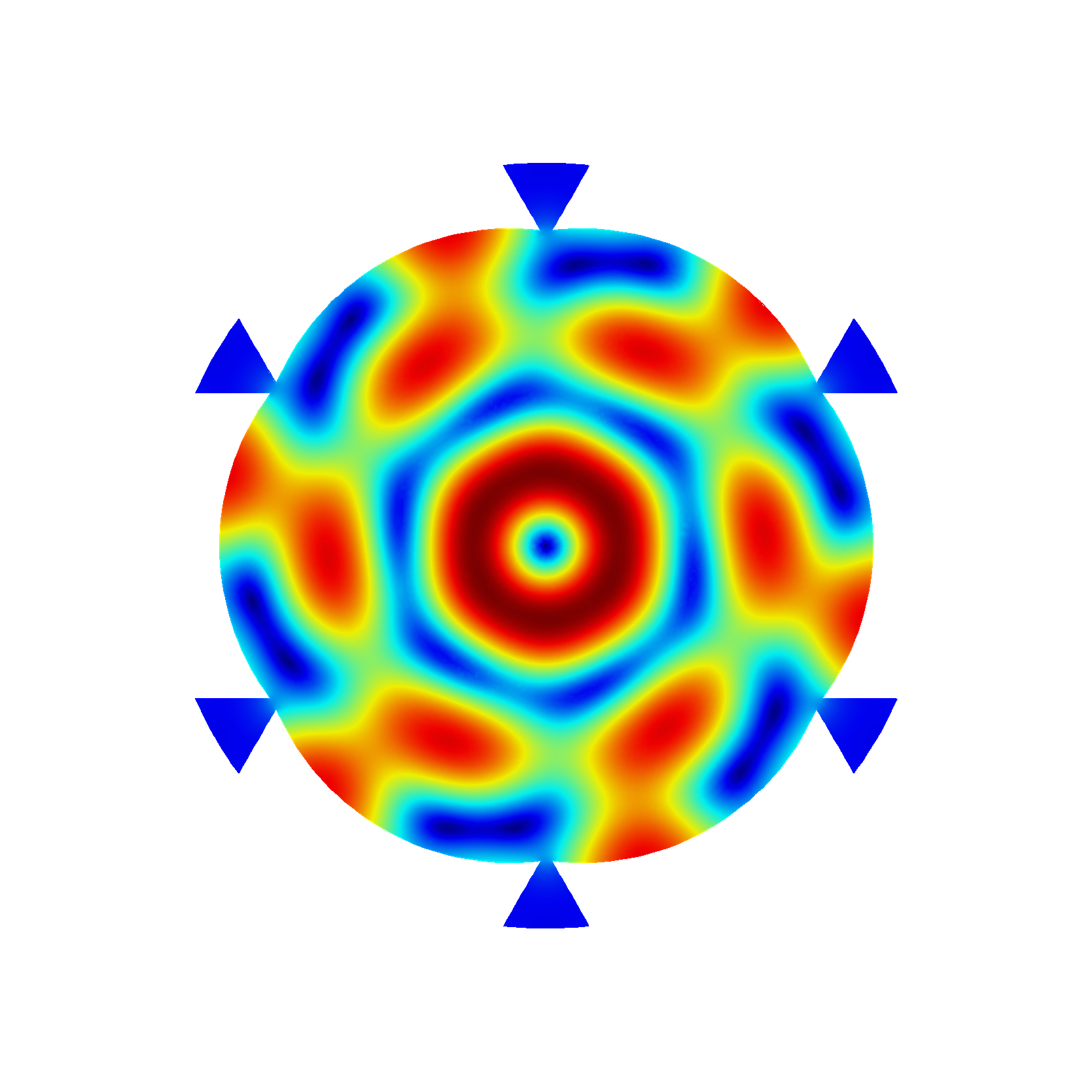


**Langevin Transducers**

**Computer**

**Signal Generator**

**Six-Channel Amplifier**

**1**

**2**

**6**

**3**

**4**

**5**


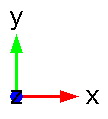


**Supplementary Figure S1. Schematic of the apparatus.** The regular-polygon array made up of six peripherally and uniformly placed acoustic sources is shown (see Methods).


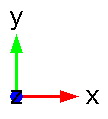

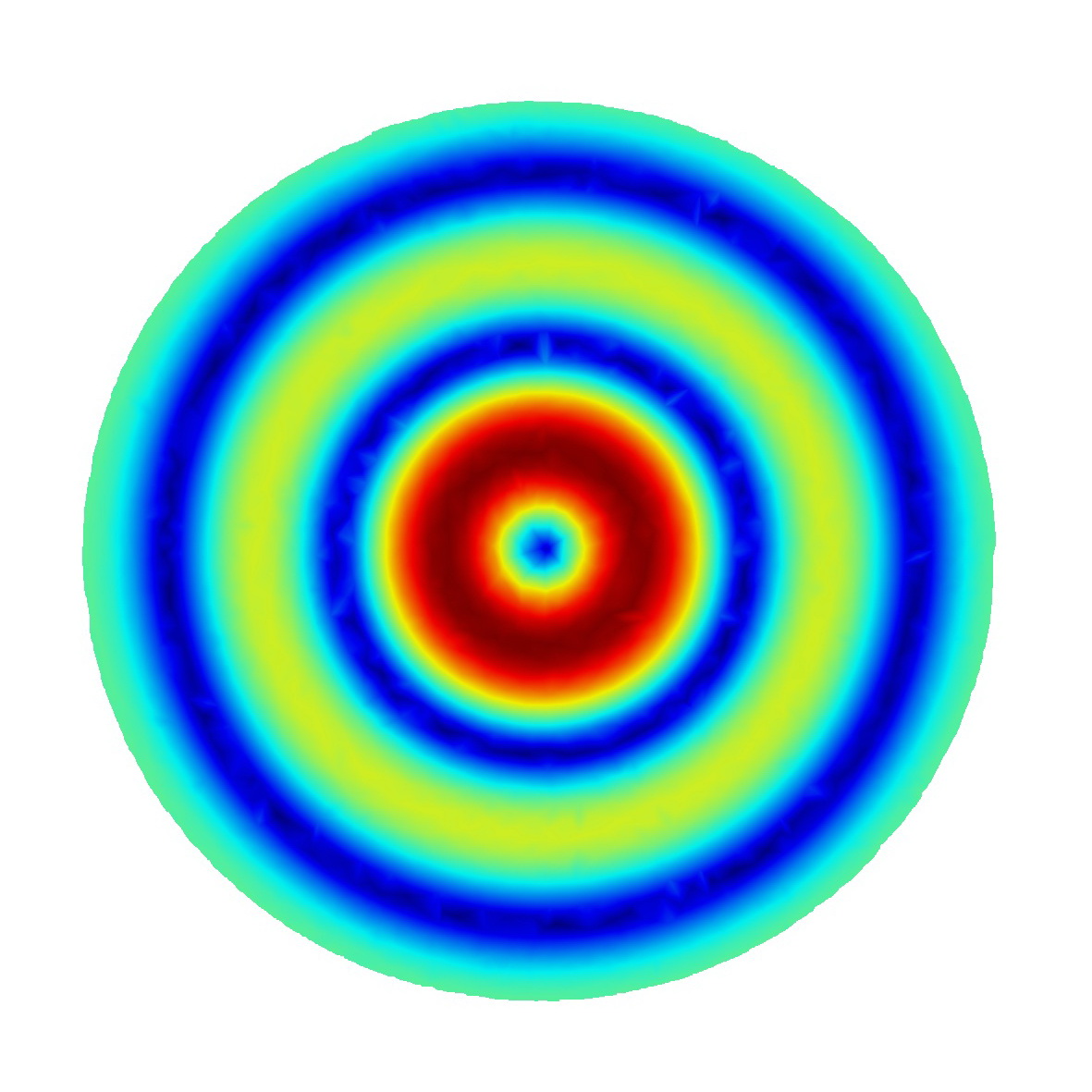

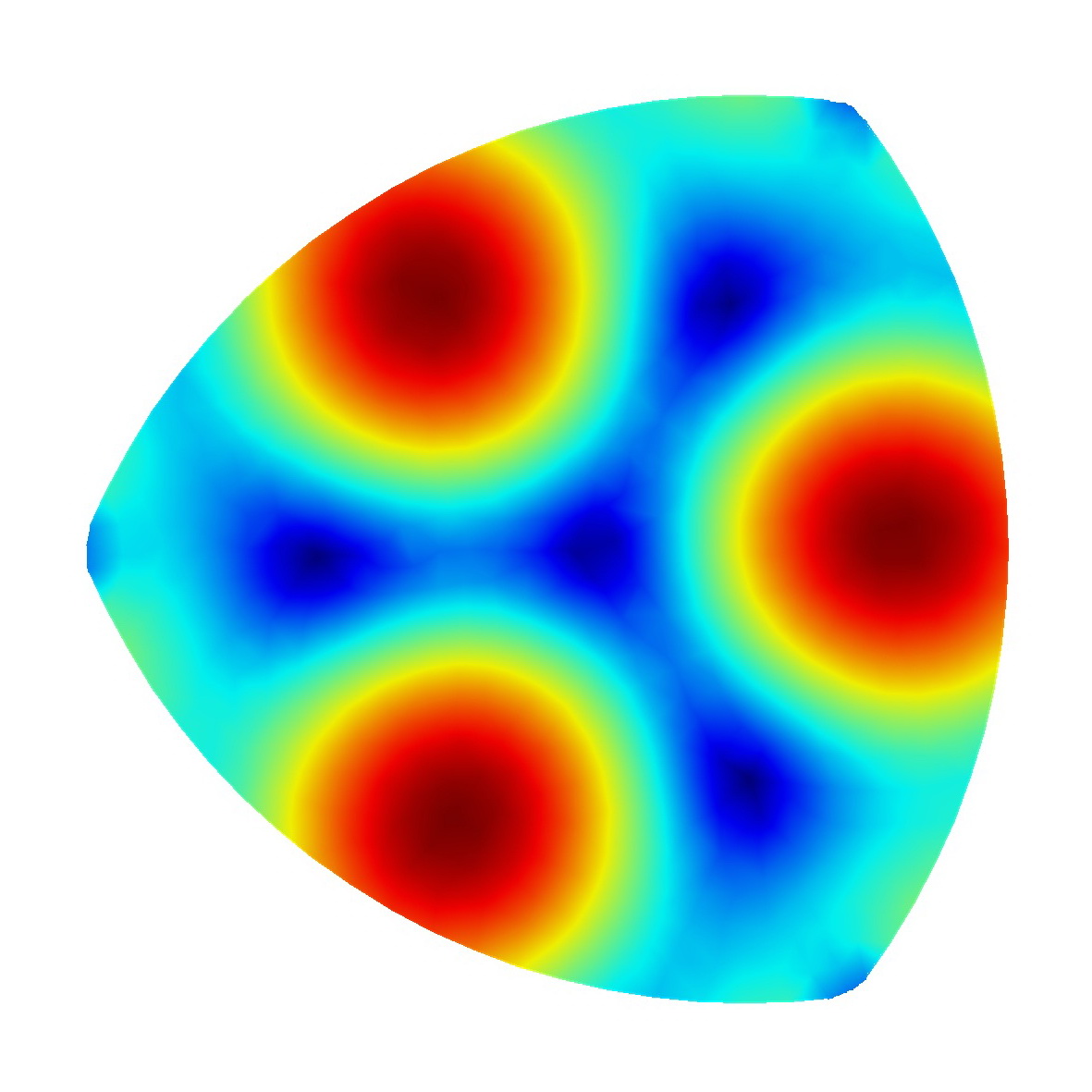

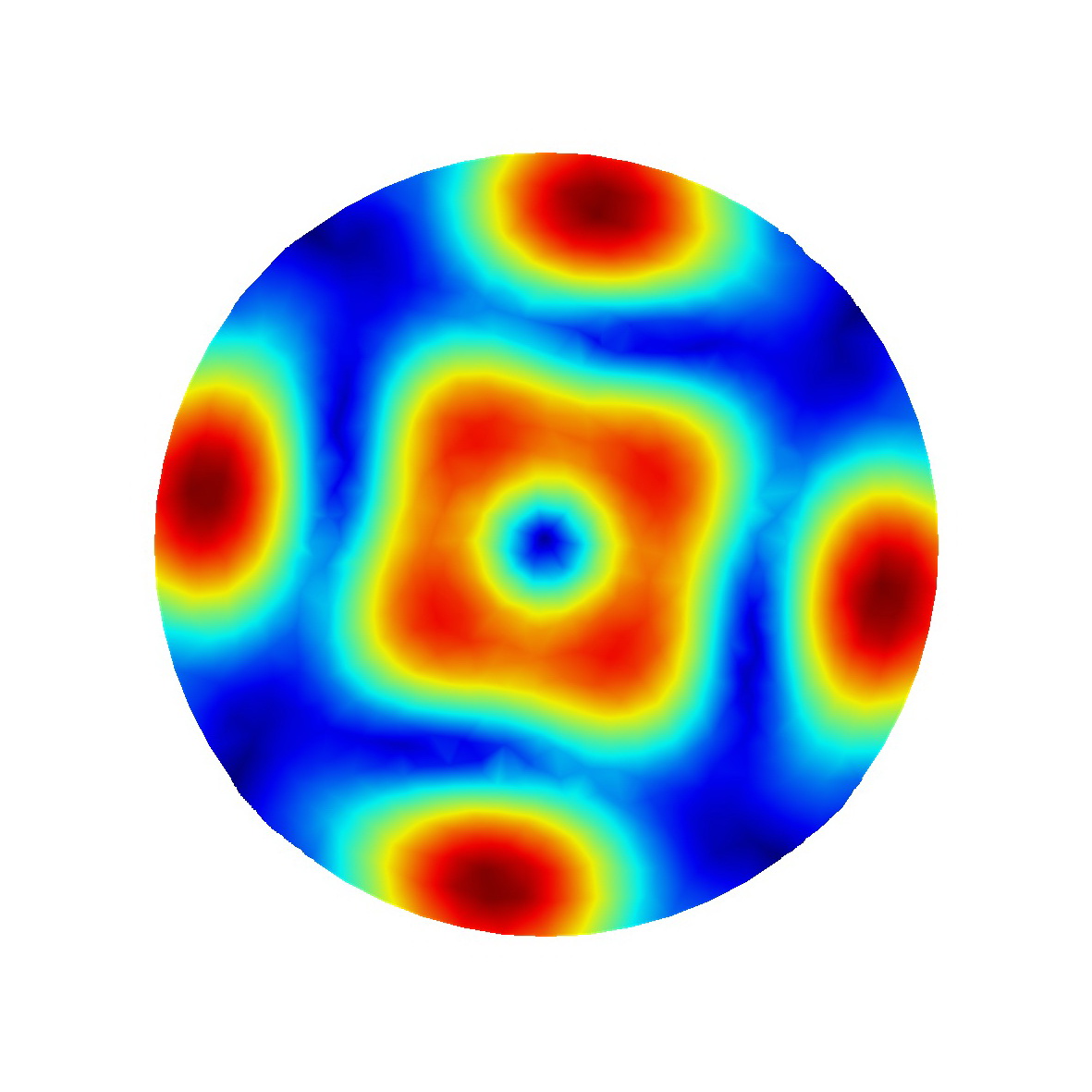

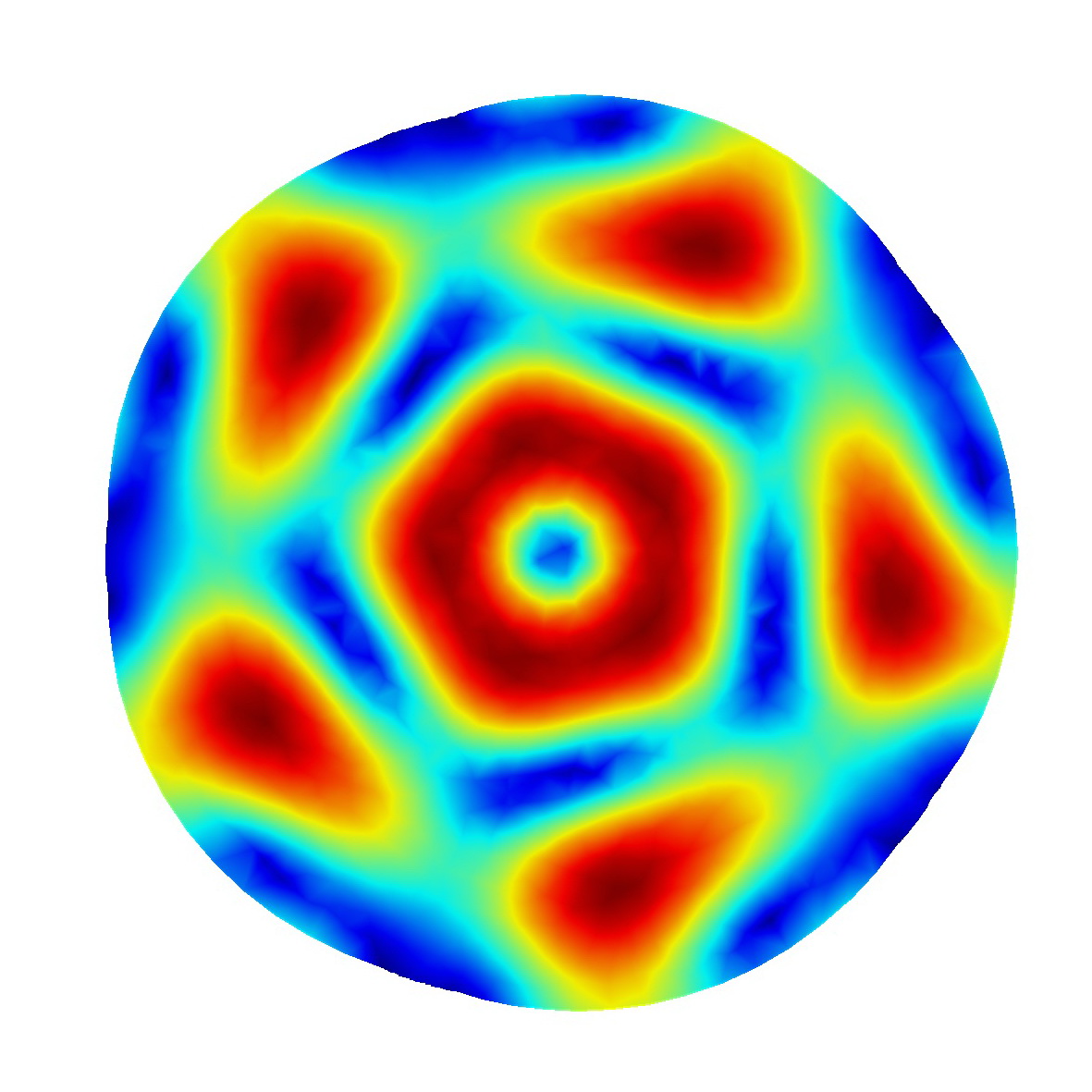

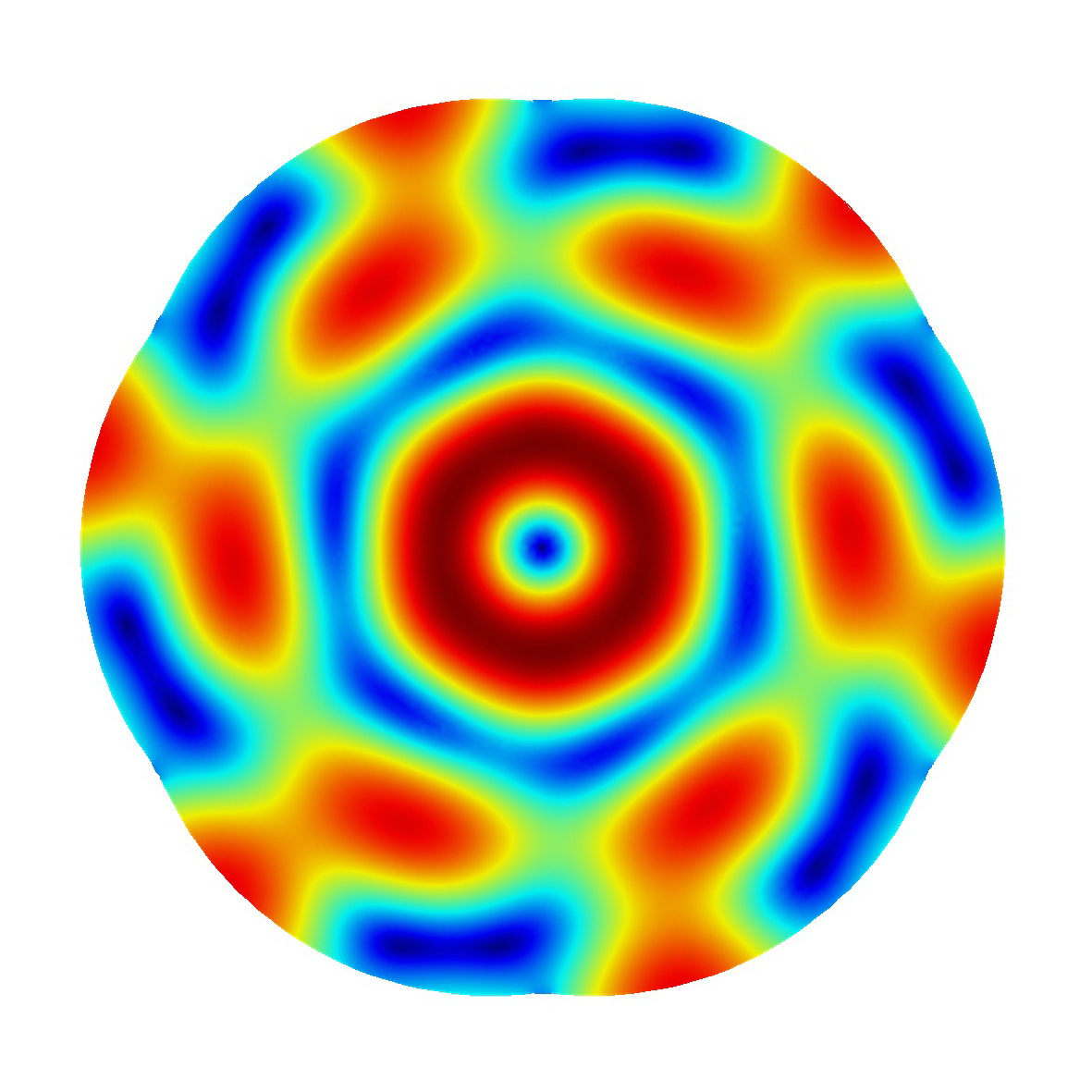


7mm

**a**

**b**

**c**

**d**

**e**

***N*=3**

***N*=4**

***N*=5**

***N*=6**

**Bessel**

**Supplementary Figure S2. The variation of acoustic pressure field with the number, *N*, of transducers.** Also a first-order Bessel-shaped field is shown.

***Schematic of the apparatus.***


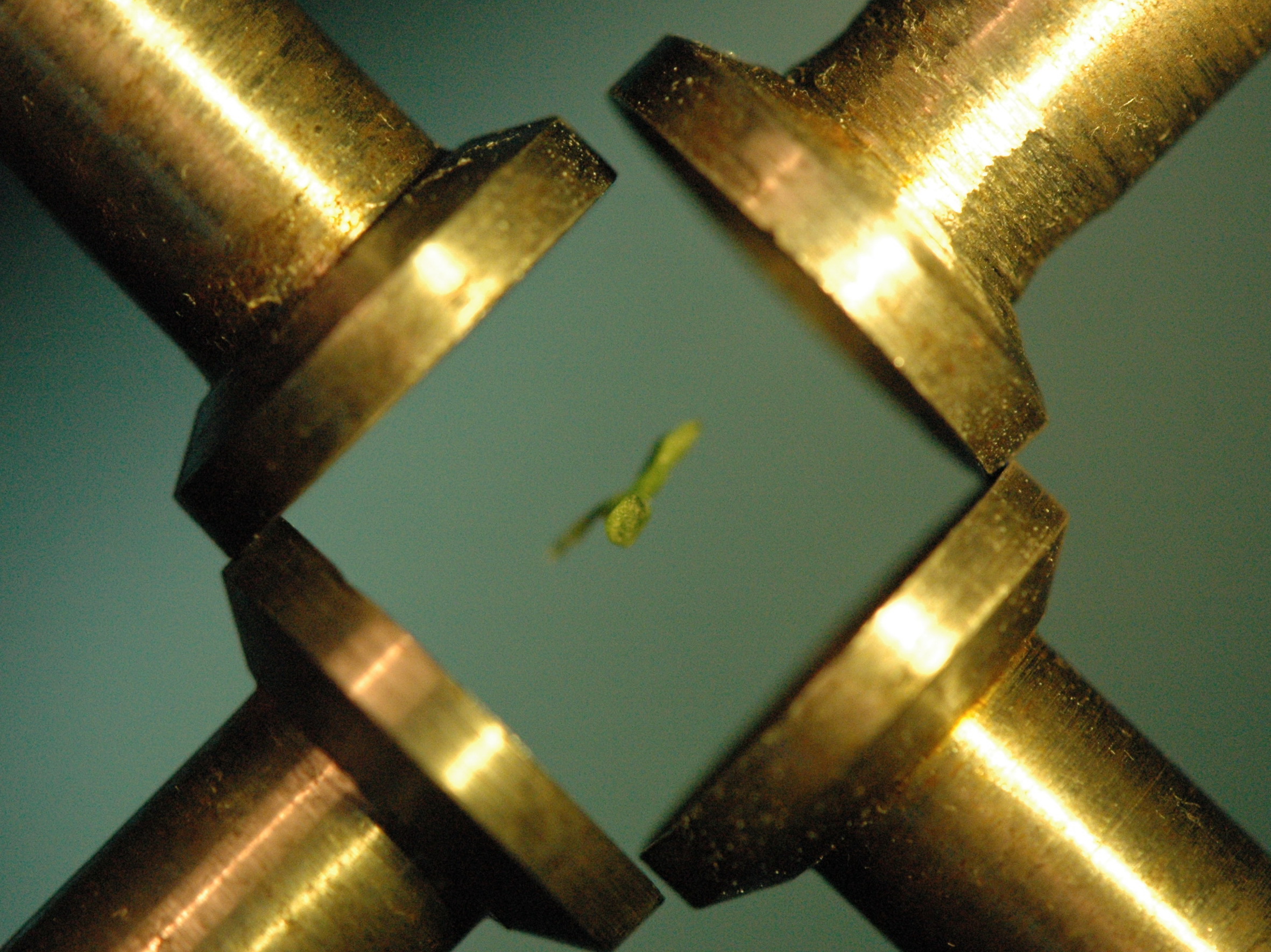

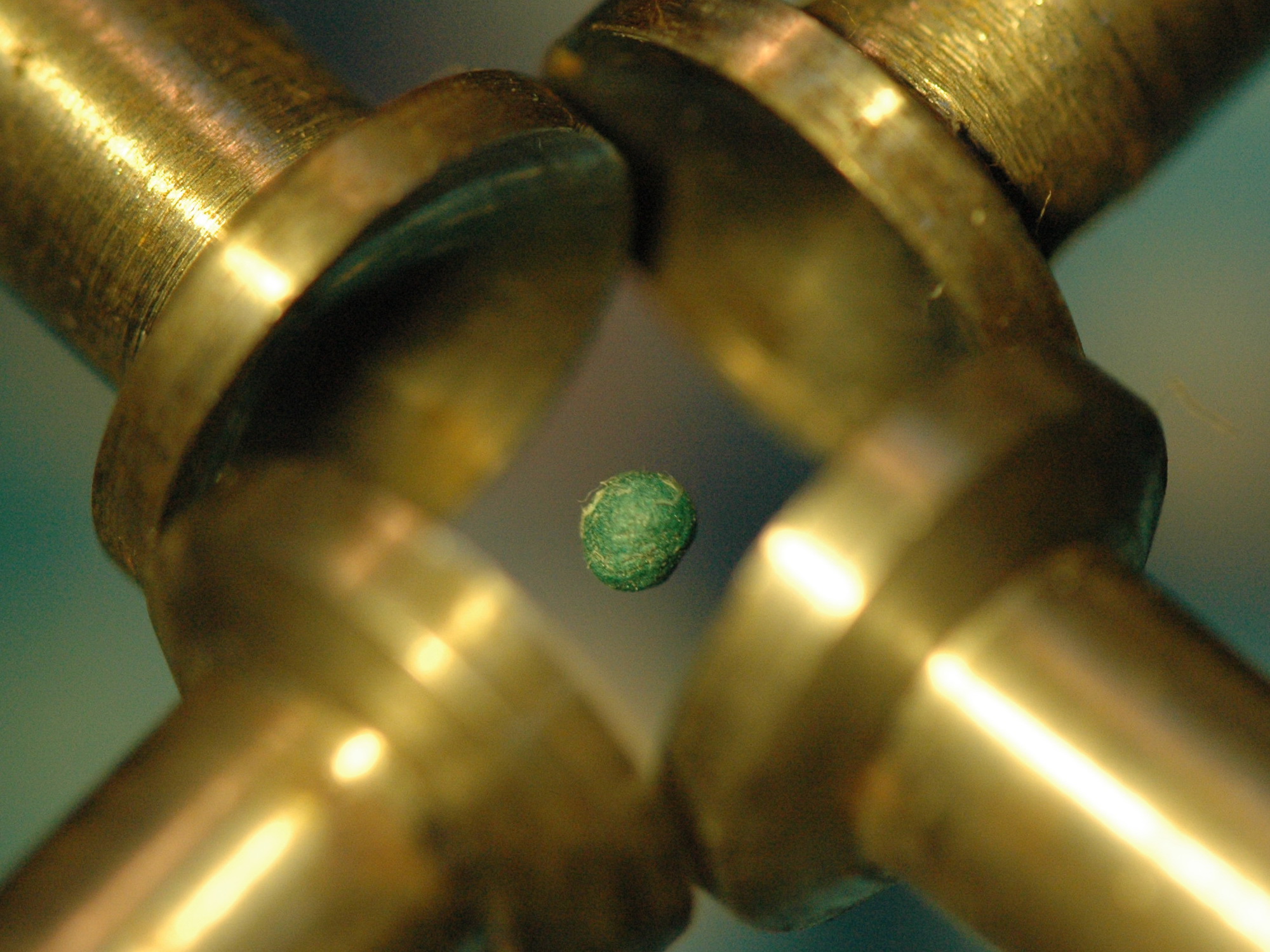

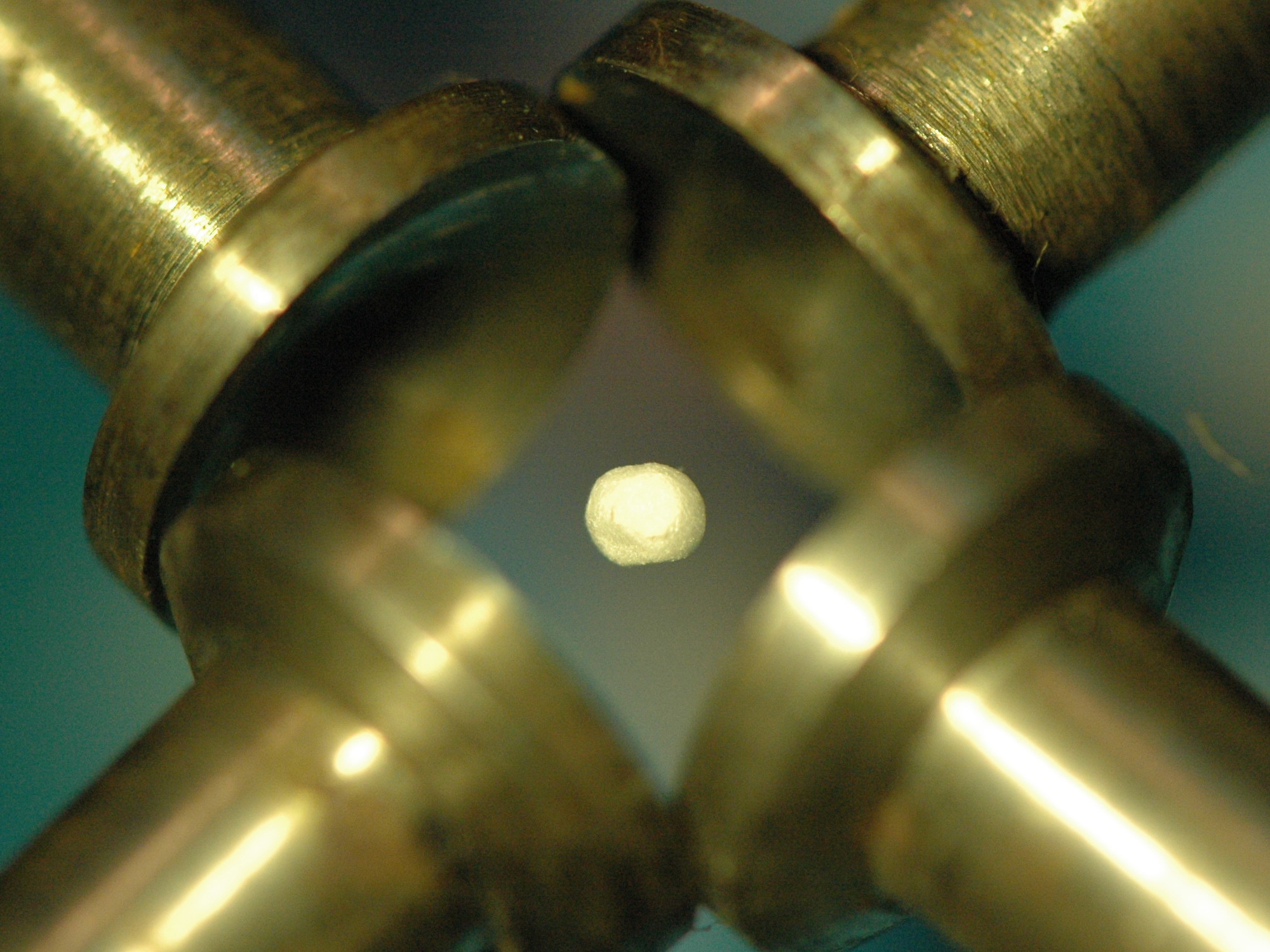

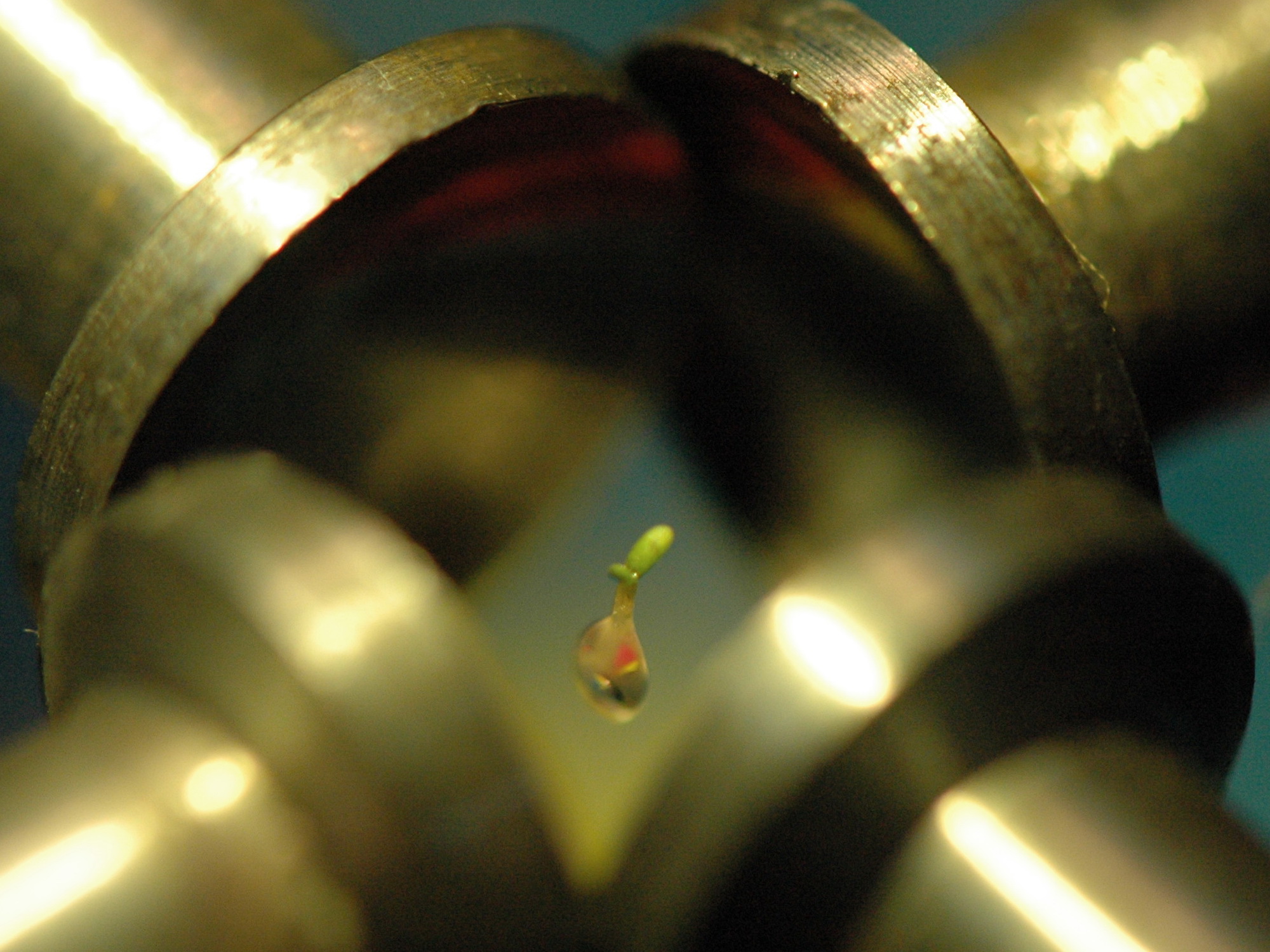


**a**

**b**

**c**

**d**

7mm

**Supplementary Figure S3. Levitating diverse objects by using the acoustic vortex field generated by four transducers.** (**a**) Expanded polystyrene sphere. (**b**) Plasticene sphere. (**c**) Vegetable seedling. (**d**) Twig.


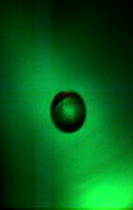

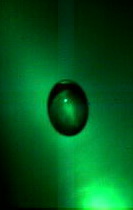

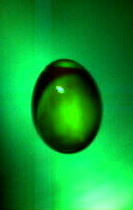

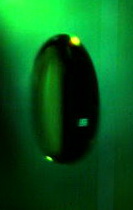


1mm


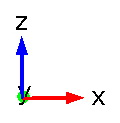


**Supplementary Figure S4. Equilibrium shapes of different sized water droplets in the central trap of acoustic vortex field.**
